# Commentary: Association of Breast Milk Fatty Acids With Allergic Disease Outcomes—A Systematic Review

**DOI:** 10.3389/fped.2018.00094

**Published:** 2018-04-09

**Authors:** Chad A. Logan, Jon Genuneit

**Affiliations:** ^1^Institute of Epidemiology and Medical Biometry, Ulm University, Ulm, Germany; ^2^‘In-FLAME’ the International Inflammation Network, World Universities Network (WUN), Ulm, Germany

**Keywords:** human milk, fatty acids, wheeze, asthma, allergic diseases

We were pleased to read the systematic review of studies investigating associations between breast milk fatty acids and allergic disease outcomes by Waidyatillake et al. (1). A comprehensive overview of systematic reviews in allergy epidemiology (2) up to 2014 has identified several systematic reviews on the association of fatty acid intake with allergic disease risk (3–8). We are aware of two further systematic reviews on this topic published in 2015 (9, 10). While these previous reviews have largely synthesized evidence on fatty acid supplementation, also during lactation, only one of them has explicitly synthesized studies ascertaining evidence on breast milk fatty acid levels—but only for asthma as an outcome (8). We thus hope that this systematic review by Waidyatillake et al. will help to spur interest on this potentially important topic.

We have published an original study (11) after the search period of the most recent systematic review which offers additional insight and may thus provide further guidance for future investigations. In our study (11), we investigated associations between breast milk fatty acid composition and several wheeze phenotypes as well as asthma diagnosis up to age 13 years. Here, we addressed several limitations implicated by Waidyatillake et al. (1) as cause for further study (i.e., large sample size, analysis of diverse outcomes, comprehensive statistical analysis, and adjustment for multiple potential covariates or confounders but not for potential mediators). We also improved upon previous studies by employing statistical methodology to account for constant-sum constraint (11), a potentially serious issue when analyzing concentration data (12, 13).

As in the majority of the studies reviewed by Waidyatillake et al. (1), we identified no convincing evidence of association between fatty acid composition and childhood respiratory outcomes (11). Furthermore, we showed that previously reported significant associations for omega-3 and omega-6 fatty acids may have been overstated due to spurious correlation between fatty acids which was not accounted for (11). Yet despite our null findings, we also do not believe that this is the end of the story. Further study of breast milk fatty acids may shed light on potentially more complex relationships between breastfeeding and disease.

Since our results diverge from previous studies likely due to different statistical methodology, we believe a good first step could be to re-examine existing data using simple statistical methodology similar to our own (11). As the sum concentration of all fatty acids within any breast milk sample is bound at 100% of total fat weight, known as the constant-sum constraint, analysis of raw data may often result in spurious correlations (12, 13). Interestingly, though these analytical methods for compositional data analysis have been used extensively in investigations of bovine milk (14), few previous studies of human breast milk constituents have truly accounted for compositionality (15). Results from appropriate re-analyses of existing data would serve to test the validity of previous findings. Moreover, they could be used to identify correlations between fatty acid constituents which may be more meaningful to infant and childhood health and disease outcomes than total fat proportions of single fatty acids or fatty acids grouped based on chemical similarities (like grouping all omega-3 fatty acids). Importantly, we show that associations may be attenuated toward the null if negatively correlated (or non-correlated) fatty acids are grouped and analyzed as such (11).

In addition, breastfed children often constitute their own specific subgroup who may be more likely to have healthier and more educated mothers than children who were never breastfed or were breastfed for only a short period of time (16). To overcome this potential selection bias, it may be important to investigate those children not receiving breast milk alone or at all. In early infancy, before introduction of solids, infant formula will be their source of nutrition. Thus, future studies could embark on the strategy of investigating the association of fatty acid profiles of both breast milk and infant formulas forming the diet of an unselected study population with allergic outcomes.

Furthermore, fatty acid concentrations in breast milk have been shown to vary over the course of lactation (17). Infant formula may provide a more stable fatty acid profile which may allow researchers to identify associations which are difficult to observe in breast milk. Following the idea that the source of fatty acids could be extended from pure breast milk to formula, future investigations should also pay attention to the evidence of intervention studies on fatty acid supplementation in pregnancy, during lactation, or during infancy. Obviously, timing of exposure is an important issue. Moreover, for maternal supplementation, maternal uptake and secretion into breast milk can lead to variation across mothers. Also, child uptake of fatty acids may differ by a various factors associated with infant feeding such as timing, frequency, and maternal diet. Therefore, another approach to exposure assessment may be to directly measure fatty acid levels in infant serum. However, this may be limited due to practical and ethical reasons in small infants but could be easier and potentially informative in animal model settings.

Finally, early infancy is a particularly important period for development of the immune system and of the gut microbiome which may potentially be associated with childhood atopic outcomes. Therefore, we agree with the current review and believe future studies should add to ours by analyzing fatty acids and other constituents in colostrum which may be differentially associated with disease outcomes.

To move forward, we suggest (i) building on the existing systematic reviews, (ii) employing the centered log ratio transformation to overcome spurious correlation, (iii) considering alternative ways of grouping fatty acids, (iv) reducing selection bias by sampling infant formula, (v) further investigating other forms of exposure assessment (upstream as maternal fatty acid supplementation, downstream, or as part of animal models), and (vi) bearing in mind timing of exposure.

## Author Contributions

Both CL and JG have conceived and written this commentary.

## Conflict of Interest Statement

The authors declare that the research was conducted in the absence of any commercial or financial relationships that could be construed as a potential conflict of interest.
